# Are we interconnected? A qualitative study on the role and perception of different actors on malaria social behaviour change interventions in rural Mozambique

**DOI:** 10.1186/s12936-020-03485-1

**Published:** 2020-11-23

**Authors:** Liliana de Sousa Pinto da Fonseca, Jorge A. H. Arroz, Maria do Rosário O Martins, Zulmira Hartz

**Affiliations:** 1 Plataforma Inter-Religiosa de Comunicação para Saúde-PIRCOM, Maputo, Mozambique; 2 Mozambique Medical Council, Maputo, Mozambique; 3 Global Health and Tropical Medicine, GHTM, Instituto de Higiene e Medicina Tropical, IHMT, Universidade Nova de Lisboa, UNL, Rua da Junqueira 100, 1349-008 Lisbon, Portugal

**Keywords:** Perceptions, Institutional and community actors, Social and behaviour change, Malaria, Qualitative study, Mozambique

## Abstract

**Background:**

Interconnecting institutions (health and education sector) and community (through a network of community structures) in social and behaviour change (SBC) activities can add value in an effort for malaria prevention towards a long-term objective of elimination. This approach has been implemented since 2011 in some rural districts of Mozambique. The objective of this study is to describe the perceptions of community and institutional actors on malaria prevention interventions in rural Mozambique.

**Methods:**

A descriptive qualitative study with a constructivist research paradigm was conducted in October 2018 in two rural districts of Zambezia Province with high malaria burden in Mozambique. Key-informant sampling was used to select the study participants from different actors and layers: malaria community volunteers, health professionals, non-governmental actors, and education professionals. In-depth interviews (IDIs) and focus group discussions (FGDs) were used to explore the perceptions of these actors. Classic content analysis looking for themes and semantics was used, and saturation guided the sample size recruitment.

**Results:**

A total of 23 institutional actor IDIs took place, and 8 FGDs were held. Four themes emerged from the content analysis: (1) organizational and functional aspects; (2) knowledge about malaria; (3) perception of institutional actors on SBC and community involvement; and, (4) perception of institutional actors on the coordination and leadership on SBC malaria interventions. Community structures were well organized, linked to the health sector and operational, with good knowledge of malaria prevention. Education sector (school teachers) links with the health sector were in some cases good, and in other cases, non-existent. The importance of SBC interventions for malaria control was recognized by health actors, although the activities are delegated to non-governmental institutions. Domestic budgetary allocation constraints, quality of intervention and lack of SBC standard indicators were also identified by health actors as aspects for improvement.

**Conclusions:**

Community structures, volunteers and primary school teachers have good knowledge on malaria prevention and regularly sensitize community members and students. Institutional health actors and partners recognize their role on malaria prevention activities, however, more interconnection is needed at different levels.

## Background

Sub-Saharan African countries carry most of the worldwide malaria burden, accounting for more than 90% of cases and deaths [1]. Mozambique is one of the leading countries contributing to this burden, with 4% of the share of the global estimated malaria cases and deaths [1].

The Global Technical Strategy for Malaria 2016-30 (GTS 2016-30) established ambitious goals and targets for the period, and at least 90% of malaria mortality and incidence is expected to be reduced by 2030 when compared to 2015 levels [2]. A strategic framework to ensure programme alignment and implementation was developed, with three pillars and two supporting elements [2]. One of the elements is to strengthen the enabling environment [2] by several activities, including of paramount importance, multisectoral collaboration, empowerment of communities, and engagement with non-governmental organizations. The engagement of the Ministry of Education (and other ministries), and close collaboration with community leaders and non-governmental partners are crucial for success [2].

To have all community and institutional actors trained in malaria prevention and approaches of social behaviour change (SBC) can add value to malaria prevention interventions [3]. Health professionals, health community workers, community leaders, teachers, and malaria officers are considered the greatest influencers on malaria prevention practices [4]. SBC activities are implemented with a large number of community health workers and volunteers who are considered agents of SBC, playing a key role in formal health services, and a link between health/social services and the community [5].

In 2011, with the financial contribution of the Global Fund to Fight AIDS, Tuberculosis, and Malaria (Global Fund), Mozambique started to implement a collaborative malaria project, a collaboration between the National Malaria Control Programme (NMCP) and civil society partners. The project was aligned with the country’s strategic plan, filling the implementation gaps that the NMCP could not reach. Among other activities, the project scope was to train community volunteers and primary school teachers to sensitize community members and students in their geographical area of action. Other activities took place, such as promoting coordination meetings between health units and community structures, broadcasting malaria preventive messages through community radios, and distribution of bed nets.

Social Ecological Model (SEM) was implemented as the theoretical framework for this study. The SEM considers individual behaviour as the product of multiple individual, social and environmental influences, and it combines individual change in order to influence social context in which the individual operates [6]. According to this model, working with community actors and institutions (e.g., those in health and education) results in more significant change in individual and community behaviour [3, 7]. This study was conducted to understand the interconnections between institutional and community actors on malaria prevention activities. The aim was to describe the perceptions of community and institutional actors on malaria prevention interventions in rural Mozambique.

## Methods

### Qualitative approach and research paradigm

A descriptive qualitative study was conducted in October 2018 in two districts (Namacurra and Nicoadala) of Zambezia Province, in Mozambique. A constructivist research paradigm was used to allow for interactive and in-depth exploration of perceptions among different actors.

### Context

Nicoadala and Namacurra are districts of Zambezia, the second-most populous province of Mozambique, located in the central region. In Zambezia, malaria prevalence in children under 5 years old is 44% [8], and 65% of the population has easy access to a health facility, i.e., less than a 30-min walk [9]. The illiteracy rate is 54%, being more prevalent in women (72%) than men (34%). Both districts were selected by researchers and provincial health authorities based on pragmatic criteria: high malaria incidence (both with more than 250 cases per 1000 inhabitants); having benefitted from malaria prevention SBC interventions led by implementing partners funded by the Global Fund, and resulting in significant case reduction from 2016 to 2018 [10–12]; and, easy access (fewer than 100 km from the capital).

### Sampling strategy

Key-informant sampling was used to select study participants, looking at different actors and layers: community volunteers, health professionals (from central, provincial and district levels), non-governmental actors playing a role on malaria interventions, and education professionals. Key informants provided leads to other key informants (i.e., snowball sampling). Saturation was the criteria for deciding when no further participant recruitment and interviewing was necessary.

### Data collection instruments and technologies

Two interview guides were developed, for in-depth individual interviews (IDIIs) and focus group discussions (FGDs). IDII was used for health professionals and non-governmental actors. FGD was used for education professionals and community volunteers. For health professionals, non-governmental actors and education professionals, the guides were developed and conducted in Portuguese. For community volunteers, the FGD guide was developed in Portuguese, tested in the local language and after necessary corrections, all FGDs were conducted in the local language. All interviews were audio-recorded and transcribed. The interviews occurred in October 2018.

### Units of study

Two units of study were considered: community actors and institutional actors. Community actors comprise community volunteers involved in malaria prevention sensitizations; these volunteers are organized into community structures. The community structures, composed of at least 15 volunteers, are trained (by non-governmental partners) on malaria knowledge and then sensitize the community in their geographical area. Institutional actors comprise health professionals (central, provincial and district levels) involved in malaria activities (promotional, prevention, diagnosis, treatment), non-governmental actors involved in malaria prevention activities, and education professionals: primary school teachers trained on malaria prevention (by non-governmental partners) and transmitting the knowledge to their students during classes.

### Data processing and analysis

FGDs were translated from the local language (*Chuabo*) to Portuguese and then transcribed. Data were translated to English. Two researchers, trained in qualitative methods, analysed the transcripts and developed themes and codes based on frequencies, common word search, identification, and classification of themes and semantics (connections between themes in the text). Transcripts were coded independently. In cases where there were discrepancies in coding, the researchers re-analysed the transcript together to reach a consensus. The identified codes and themes were analysed using NVivo 12 software. The researchers could triangulate various sources to verify consistency and improve the validity of data. Saturation guided the quantity and quality of information analysis.

### Techniques to enhance trustworthiness

The fact that some of the study participants were direct actors working on SBC interventions could be a source of bias. However, being key informants was also a strength of the study. To minimize bias being introduced by these actors, the interviewers were not involved in SBC interventions and had a very good background on qualitative interviewing techniques conducting the interviews in a very specific manner. Additionally, probe questions were introduced for later triangulation of the responses.

## Results

Table 1 summarizes the main points collected in this study, organized by approach and target group. A total of 17 individual interviews of institutional actors took place. Of these, 5 were from central level, 4 from provincial level, and 8 from district level. A total of 7 FGDs were held. Of these, 4 were with FGD community structure volunteers (each FGD had 8–12 volunteers), and 3 with primary school teachers.

**Table 1:** Qualitative approach, target groups, and main results

Actors	Central (donors, IP, MoH)	Provincial (IP, PHA)	District (DHA, CA, CHW, CS, ST)
Approach	Individual interviews	Individual interviews	Individual interviews—DHA and CHW FGD—CA, CS and ST
Target group (number of interviews)	MOH National Malaria Control Programme (1) Implementation partner (3) Donor (1)	Provincial Health Directorate Provincial health staff (2) IP (2)	District health staff: DHA staff—Namacurra (3), Nicoadala (3) CHW—Namacurra (1), Nicoadala (1) Community actors: CS working on SBC activities—Nicoadala (2), Namacurra (2) Primary ST working on SBC—Nicoadala (2), Namacurra (1)
Responsibilities	Update the SBC strategies and budget allocation per province Coordinate with donors and central IP	Design the provincial work, budget, and implementation plans	Field implementation
Main result: CS and ST: Organizational and functional aspects			CS and ST have regular meetings with DHA
Main result: CS and ST: Malaria knowledge	Design of the training curriculum	Training and monitoring/supervision	CA have good knowledge about malaria (mode of transmission, signs and symptoms, and where to seek treatment) More information is needed about the importance of IPTp
Main result: Perceptions about SBC activities and community involvement	SBC intervention is the key to malaria prevention and control	SBC intervention is very important	
Main result: Perception about coordination and leadership of the SBC malaria intervention	Lack of central level (MOH) commitment to enable them to take on the technical leadership of the action plans Involving communities at the grassroots is challenging SBC activities are not prioritized in terms of budget allocation	Quality of SBC interventions should be a focus area Lack of standard SBC key indicators Communication and coordination are the key for the success of SBC activities (there is a need for more coordination between the donors and all sectors—for example, education—not just the MoH)	

*CA* community actors, *CHW* community health workers, *CS* members of community structures, *DHA* district health authorities, *FDG* focal group discussion, *IP* implementing partners, *IPTp* intermittent presumptive treatment in pregnant women, *MoH* Ministry of Health, PHA, Provincial Health Authorities, *SBC* social behavioural change, *ST* school teachers

### Themes from content analysis

Four themes emerged from content analysis: (1) organizational and functional aspects; (2) community structures and school teachers’ knowledge about malaria; (3) perception of institutional and community actors on SBC and community involvement; and, (4) perception of institutional actors on the coordination and leadership on SBC malaria interventions.

### Organizational and functional aspects of community structures and school teachers regarding malaria prevention

Community structures have regular monthly meetings with health facilities to discuss malaria issues and possible solutions. A summary report is written after each meeting to allow for follow-up. Most of the participants reported that they have a work plan and communication materials, such as malaria flipcharts and flyers, and also have T-shirts, caps, and *capulanas* (a traditional type of sarong considered a complete piece of clothing, that can either be used as a wrap-around skirt, dress or become a baby carrier) printed with malaria preventive messages and images, which serve as their identification as malaria community volunteers.

“We meet with the health facility once a month and we have to write a summary report after each meeting. We have a work plan, which is divided into groups, so that this group will work this week and another group will work another week. At the meeting, each group brings the difficulties encountered in the area where they worked. The material that was given to us was a blue *capulana* with illustrations and pamphlets, which we extended and used to explain. We have flipcharts, T-shirts, identification caps, capulanas, pens, and notebooks.”

FGD1_CS Nicoadala

During the FGDs, some school teachers mentioned that there was a very strong and positive coordination between their malaria activities, the health facility, and health professionals. School teachers reported that they usually meet with the health facility on very specific dates every month. Participants showed their SBC materials such as: flyers and facilitator manual with malaria messages. They also requested more SBC materials, such as large-sized posters with illustrative images to facilitate visibility for children, especially those sitting at the back of the room.

“…We have met yes, monthly. Three times per month. We don’t have a schedule. It has been a random process and when there was an opportunity, we met. Well, regarding the coordination with the health facility, it is very positive. At some point on this exchange of information, we get information on how malaria is transmitted and how to prevent it. We have used some leaflets to show some images in the lectures, orally we have also spoken explaining to the children and we have also the facilitator’s manual. We need large posters to make it easy for kids who sit at the back to see.” FGD2_PR Nicoadala

In contrast, other school teachers reported that they never had a meeting with the health facility or staff to discuss malaria. They just worked with students and their caregivers and had a malaria flipchart to work with.

“….. No. We were only trained to reach students and their caregivers. We don’t have any link with the health facility or health staff. As we said, we only use the flipcharts.”

FGD3_PR Namacurra

### Malaria knowledge of community structures and school teachers

Community structures volunteers and school teachers demonstrated relatively good knowledge about malaria, its mode of transmission, signs and symptoms, and where people should go as soon as they become ill.

“…..We know that malaria is a disease that kills but also has a cure, we can medicate, or we can prevent by using these mosquito nets. In case you get the disease, you have to go to the health facility immediately to actually detect the disease. Just showing the symptoms (headaches, cold, joint pain, diarrhoea, and vomiting) is not enough because malaria manifests itself in various ways depending on one’s body….”

FGD2_PR Nicoadala

“…Malaria is transmitted through the mosquito bite. An infected person transmits the disease to a healthy person. If not treated, malaria can be dangerous and fatal. To avoid malaria, I sleep every night under a mosquito net but not only this, I take care of my house environment (cleaning, eliminating stagnated water, and sometimes I burn some plants to avoid mosquitoes). The signs and symptoms of malaria include cold, vomit, shaking, the body is warm, headache, diarrhoea, and pain at the joints. Malaria has treatment and we need to go to hospital for treatment and there you can find the right medication. It’s important to finish all the medication course they give there.”

FGD1_CS Nicoadala

During FGDs, school teachers mentioned malaria in pregnancy and its importance. The majority of school teachers consider that the disease acts equally at all ages but in pregnant women, children and the elderly there is a need for careful consideration due to various factors, such as immunity. However, they acknowledged that there are still challenges regarding the adherence to the malaria intermittent presumptive treatment in pregnant women (IPTp), as many still need more information about the importance of taking IPTp for a healthy pregnancy and for the baby.

“…it has to do with immunity, because the pregnant woman shares her body with two people, she is a little weak, while the young man is there alone has nothing to join inside and always comes out on top. The old are also a little weak in immunity (…) pregnant women when they have malaria, if in the first months they may have abortion scares.”

FGD1_PR Nicoadala

Several respondents described bed nets as the most used preventive method. Community structures reported that bed nets have a double function as they protect from mosquitoes and other animals. Indoor residual spraying (IRS) was considered not very useful by the community because it only works inside the house and people usually sleep outside because it is cooler. Other methods of mosquito avoidance included burning plants or using green leaves from trees to drive off mosquitoes.

“…in our community, the bed net is the most used to prevent malaria (…) because it is easy to use and protects us from other insects and animals during the night. We don’t like PIDOM (IRS), it is only useful inside our house and we like to sleep outside when it is too warm. Of course, we also use our local methods such as burning plants and using the green leaves of our three to avoid mosquitoes.”

FGD1_CS Namacurra

### Perceptions of health institutional actors about SBC activities and community involvement

Institutional actors (implementation partners and focal points of the Ministry of Health) were unanimous in agreeing that SBC activities are important interventions for malaria control and to ensure community engagement. SBC intervention was ranked 5 out of 5 using a Likert scale with ascendant score. They also reported that community actors participate in SBC activities through a reciprocal relationship between implementation partners (non-governmental actors), community (community structures, local religious leaders, and others), and government (health sector, education sector, district government, and others).

“… Well, the SBC intervention is very important (…), through SBC we can design some strategy in how to engage the community at all levels, so this is a very key area, because this area designs the strategy to engage the community. This coordination between partners (…), community (…), and us from the system, I think the information arrives more easily to the community level.”

IDII1_PHA Zambezia

“…I give 5, because the SBC intervention is the key to malaria prevention and control, but the SBC intervention should be with all stakeholders, both among health professionals, partners, and communities for prevention (…)”

IDII1_IP Maputo

### Perceptions of institutional actors about the coordination and leadership of the SBC malaria interventions

Some challenges and barriers to community participation in SBC activities were pointed out by institutional actors such as: quality of communication of the implemented activities, the coordination at different levels of action (central and provincial level technical leadership), the lack of communication indicators that allow for measuring the results and impact of activities at provincial and district level, the limited budget allocated to communication activities, and project sustainability. Beneficiaries’ ownership (and not government or partners’ responsibility) was mentioned as a key point for sustainability of SBC interventions when there is lack of funds. They also pointed out that the focus should mainly be in the communication and coordination between all stakeholders.

“… I think what we are fighting (…) right now is the question of quality, quality of our actions in terms of SBC strategies that we are planning and implementing, how the information (key messages) are passing and spreading into the community, and not only ready to come up with community talks (…) we have to know if people really understand and have the right attitude, are they meeting what is required or not? (…) So, our big challenge is the quality of our intervention.”

IDII1_IP Zambezia

“….there has been good progress on malaria prevention and SBC activities, there is a need to have a commitment from the Ministry of Health (MoH) to enable them to take on the technical leadership of the processes that are taking place right now so that actions are more coordinated, more reality-adjusted and more effective. Involving communities at the grassroots, this is the central challenge. Empower the MoH to be able to assume this technical leadership, ensure coordination and contextualization and ownership of activities by communities.”

IDII2_IP Maputo

“…I think that starting from the principle that (…) I keep hitting the same key, I start from the principle of setting clear indicators, no institutional partner will actively engage in an activity that does not directly evaluate itself (…) But all we do so far, when we report to the health provincial directorate (DPS), there is no template to report communication activities, inclusive there is no clear standard indicators at the provincial and district level.”

IDII4_IP Zambezia

“….. The SBCC activities aren’t prioritized in terms of budget allocation.”

IDII3_IP Maputo

“…Communication and coordination are the key words for the success of the activities. We have to take into account the relation between health professionals, patients, and communities.”

IDII1_IP Zambezia

## Discussion

This study shows that community actors, represented by malaria volunteers organized in community structures, have a functional structure with regular monthly meetings and share good knowledge on malaria prevention. School teachers are involved in malaria prevention activities, presenting good and basic knowledge on malaria prevention methods to vulnerable groups (women, children and the elderly). However, the link between school teachers and health facilities is only partially established, with some school teachers with very positive links and others without any. This link can allow school teachers to be more effective by disseminating health promotion information to the students and their families, which can help with prevention against malaria and result in improved school retention. Health actors (from the MoH and implementing partners) recognize the importance of SBC interventions for malaria control and to ensure community engagement, but they also pointed out institutional coordination and leadership as challenging, mainly in three aspects: (1) quality of intervention; (2) lack of SBC standard indicators; and, (3) budgetary constraints.

**Fig. 1:**
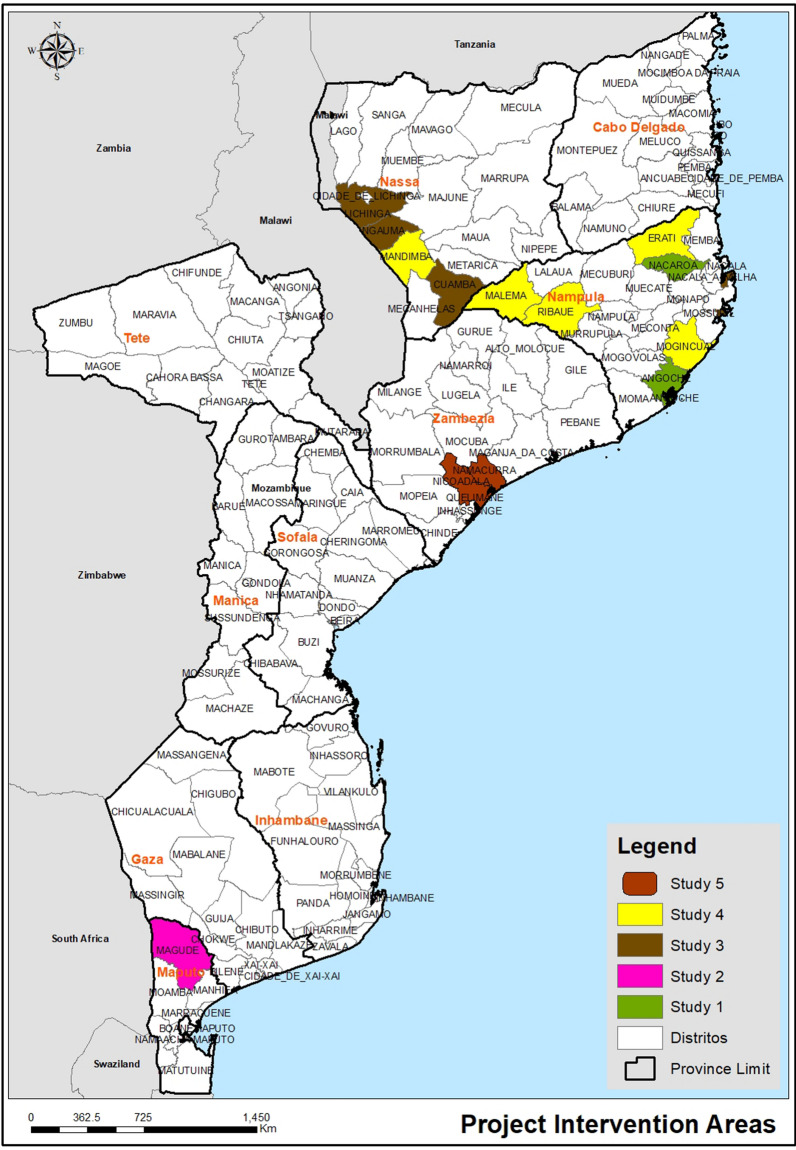
Map showing studies on the effectiveness of malaria interventions in Mozambique. Study 1, a qualitative study conducted in Nampula, a northern province, similar to Zambezia, with high malaria prevalence. The study explored the gendered decision-making matrix for malaria prevention and treatment. Study 2, a qualitative study conducted in Maputo province, Magude district, and examined community perceptions of malaria to inform elimination efforts in Southern Mozambique. Study 3, a qualitative study that collected evidence about integrating malaria education into primary school activities in Nampula (Ilha de Moçambique; Nacala Porto) and Niassa (Ngauma, Cuamba, and Chimbunila districts) provinces. Study 4 was about mobilizing communities for malaria prevention and control in Mozambique (Nampula province: Erati, Malema, Ribáuè, and Mogincual districts; Niassa province: Mandimba District). Study 5 (this study), a qualitative study that analyses the perceptions and interconnections between different actors (institutional and community) implementing SBC interventions in Zambezia province: Nicoadala and Namacurra districts

The community participation and functionality observed in this study is aligned with the SEM [3, 6, 7] and current strategies for malaria prevention and control. As per the GTS 2016-30, a close collaboration between communities and the health sector is desired and can add value in malaria prevention interventions [2–5]. Training of community structures on malaria prevention activities might ensure the community has knowledge about how to prevent malaria and how to get treatment for the disease when needed [3, 7]. Other studies found similar results (Fig. 1). A study conducted in two provinces in the north of Mozambique (Nampula and Niassa) identified community structures, trained and allocated SBC material according to their capacity, skills and needs, and demonstrated that community structures were well organized, developed a community mobilization work plan where they delivered key malaria prevention messages, and progress reports discussing the challenges encountered during meetings with the health facility [13]. In these provinces, community structures were identified as the primary source of malaria prevention information, including the correct and consistent use of mosquito nets [13, 14].

The inclusion of primary school teachers as institutional actors widened the reach of SBC intervention, showing that actors other than those from the health sector can be successfully involved in malaria prevention, which is aligned with the SEM [3, 6, 7]. A study in Thailand demonstrated that a school-based malaria prevention approach through training teachers has been widely used for malaria control with positive outcomes in the behaviour of school children [15]. A learning brief published by the Malaria Consortium revealed a similar integration process of malaria education into primary school activities in Mozambique, concluding that educational and participatory malaria sessions in schools are feasible, providing an alternative source for increasing the knowledge of both pupils and teachers [16]. However, this approach required strengthening the coordination between the health and education sectors, which in some cases were good and in others were non-existent. This different pattern of coordination might be explained by an absence of a coordination platform, such as a Memorandum of Understanding (MoU) between the Global Fund and the Ministry of Education. The MoU would result in more intervention and monitoring of the provincial and district level by central level education sector actors.

Although SBC interventions were widely recognized by the different actors as an important aspect of malaria prevention and control, the following challenges were pointed out: quality of the interventions, lack of SBC standard indicators, and budgetary constraints. For high-quality SBC interventions integrated into the malaria prevention and control plan, it is important to define target groups and behaviour-improving targets to prevent, treat and control malaria [17]. A strong perception of the need for different approaches and innovative ways to communicate is felt by MoH actors. The Mozambican NMCP is currently revising its national malaria control communication strategy to ensure different and innovative high-quality interventions and the integration of key SBC indicators can be tracked and measured at different levels of implementation [18]. Community participation can be successful once SBC interventions are adequately planned and coordinated [13]. A classic example of good and successful coordination for malaria prevention and control is the advocacy and micro-planning process of a mass mosquito net distribution campaign, where actors at different levels planned and implemented the campaign together [19].

Budgetary allocation constraints were also pointed out as being challenging, mainly due to the fact that communication activities are funded to a lesser extent and are often subjected to budget cuts when priorities have to be re-set. Low domestic budgetary allocation follows a similar pattern. For example, Mozambique’s domestic funding for the NMCP for the 2016-18 period was less than 2% of the Global Fund contribution, and less than 5% of the President Malaria Initiative (PMI/USAID) contribution [1]. In Cameroon, the National Malaria Strategic Plan (2014-2018) identified priority areas and government and partners allocated a lower budget for SBC interventions than for prevention and case management [20]. The US government allocated a higher budget for malaria case management and prevention than to SBC interventions in Mozambique [21]. For the period 2014–2019, the Mozambican NMCP allocated most of its resources, almost 79%, for medicines and commodities (mostly mosquito nets acquisition and implementation), and very few resources, 10%, for communications (SBC), media and outreach, with another 10% for programme management (including IRS and operation and coordination meetings at national and provincial level) [22].

## Limitations

This study is based on self-reported information and lived experiences. Some respondents may have mentioned some ideal perceptions or experiences unrelated to their everyday life (Hawthorne effect). However, the triangulation of data collection techniques, the use of interviewers trained in these techniques, the introduction of probe questions, the diversity of actors interviewed, and the triangulation of information among researchers allowed the potential bias to be minimized. Additionally, the study took place in only 2 out of 22 districts of Zambezia due to limitation in funding. Therefore, it is important to interpret the results with caution and without undue generalization.

## Conclusion

Community structure volunteers and primary school teachers have good knowledge of malaria prevention and they regularly sensitize community members and students. The institutional health actors and partners recognize their role in malaria prevention SBC activities, and give credit to the SBC interventions for malaria prevention and control. Although malaria prevention SBC activities are currently extended to community members and school teachers, more interconnection is needed at different levels, which could be facilitated by the MoH. The quality of interventions, lack of communication standard indicators, and limited budget allocation for SBC intervention jeopardize and condition the SBC interventions.

## Data Availability

The datasets used and/or analysed during the current study are available from the corresponding author upon reasonable request.

## Abbreviations

FGD: Focus group discussion
IDII: In depth individual interview
MoU: Memorandum of understanding
NMCP: National Malaria Control Program
PMI/USAID: President’s Malaria Initiative/United States Agency for International Development
SBC: Social and behavior change
SEM: Social ecologic model
